# Histone Deacetylase Inhibitors as Therapeutic Interventions on Cervical Cancer Induced by Human Papillomavirus

**DOI:** 10.3389/fcell.2020.592868

**Published:** 2021-01-28

**Authors:** Natália Lourenço de Freitas, Maria Gabriela Deberaldini, Diana Gomes, Aline Renata Pavan, Ângela Sousa, Jean Leandro Dos Santos, Christiane P. Soares

**Affiliations:** ^1^Department of Clinical Analysis, School of Pharmaceutical Sciences, São Paulo State University (UNESP), Araraquara, Brazil; ^2^Drugs and Medicines Department, School of Pharmaceutical Science, São Paulo State University (UNESP), Araraquara, Brazil; ^3^Institute of Chemistry, São Paulo State University (UNESP), Araraquara, Brazil; ^4^CICS-UBI – Health Science Research Centre, University of Beira Interior, Covilhã, Portugal

**Keywords:** histone deacetylase inhibitor, cervical cancer, human papillomavirus apoptosis, cell cycle arrest, senescence, tumor suppressor, HPV E6/E7 modulation

## Abstract

The role of epigenetic modifications on the carcinogenesis process has received a lot of attention in the last years. Among those, histone acetylation is a process regulated by histone deacetylases (HDAC) and histone acetyltransferases (HAT), and it plays an important role in epigenetic regulation, allowing the control of the gene expression. HDAC inhibitors (HDACi) induce cancer cell cycle arrest, differentiation, and cell death and reduce angiogenesis and other cellular events. Human papillomaviruses (HPVs) are small, non-enveloped double-stranded DNA viruses. They are major human carcinogens, being intricately linked to the development of cancer in 4.5% of the patients diagnosed with cancer worldwide. Long-term infection of high-risk (HR) HPV types, mainly HPV16 and HPV18, is one of the major risk factors responsible for promoting cervical cancer development. *In vitro* and *in vivo* assays have demonstrated that HDACi could be a promising therapy to HPV-related cervical cancer. Regardless of some controversial studies, the therapy with HDACi could target several cellular targets which HR-HPV oncoproteins could be able to deregulate. This review article describes the role of HDACi as a possible intervention in cervical cancer treatment induced by HPV, highlighting the main advances reached in the last years and providing insights for further investigations regarding those agents against cervical cancer.

## Introduction

### Cervical Cancer Associated With Human Papillomavirus (HPV)

Cervical cancer is the fourth most common cancer found in women. In 2018, it was estimated that around 570,000 women were diagnosed with cervical cancer worldwide and about 311,000 women died from the disease (Arbyn et al., 2020). Cervical cancer has its highest incidence in low-income regions such as Africa, Latin America, and the Caribbean (Ferlay et al., 2019). The ranking of cervical cancer in each country (all cancer sites in women), stratified by number of cases in all ages. Arbyn et al. (2020) related the frequency of cervical cancer as three times higher than all cancers in women of all ages.

Martel et al. (2020), based on the GLOBOCAN database for 2018, described that is estimated by us to be 690 000 new cases (age standardized incidence rateŰASIR) of 8.0 cases per 100,000 person-years, focused on HPV infection.

The HPV virus belongs to the Papillomaviridae family and can be grouped into five different genera (alpha, beta, gamma, mu, and nu), according to the differences between the DNA sequences (Choi and Park, 2016). The alpha-papillomavirus genus includes genotypes that are responsible for infections at the level of the genital mucosa, while the remaining types are characterized by their ability to infect at the cutaneous level (Bouvard et al., 2009).

The different genotypes that infect mucous membranes can also be classified according to their oncogenic potential. Low-risk groups give rise to benign lesions, such as condylomas, while high-risk groups are considered oncogenic and cause persistent infection (reviewed by Mesri et al., 2014). HPVs have been classified in carcinogen categories as carcinogenic (Group 1), probably carcinogenic (Group 2a), possibly carcinogenic (Group 2b), not classifiable (Group 3), or probably not carcinogenic (Group 4) (reviewed by Schiffman et al., 2009). Considerable evidence has shown that all cases of cervical cancer are caused by persistent infections of specific human papillomaviruses (IARC, 2021). HPV 16 and HPV 18 are clearly powerful cervical cancer agents, which are present in precursor lesions associated with an extremely high absolute risk of CIN3 and cancer (IARC, 2021). Past studies have already described HPV infection as the main risk factor in the development of benign and malignant cervical lesions (zur Hausen, 1977; Gissmann et al., 1984). Between 1983 and 1984, oncogenic HPV types 16 and 18 were identified (zur Hausen, 2011) by a German virologist and Nobel Prize recipient (in 2008), Professor Harald zur Hausen.

HPV infection accesses and infects the basal cells through micro-abrasion; the virus enters the basal layer and establishes a long-term infection in these dividing cells (McBride and Warburton, 2017). The infection begins in the transformation zone of the cervical epithelium, located between the pavement epithelium and the columnar epithelium exposed by this micro-lesion (Herfs et al., 2012), and in DNA samples from cervical specimens, which verified the majoritarian presence of HPV16 in the ectocervical/transformation zone in premalignant (CN1), junctional epithelium (CIN1), and epithelial malignant lesions (CIN 2/3 and squamous cell carcinoma) and glandular malignant lesions (adenocarcinoma and invasive adenocarcinoma). A lower frequency of other HPV types was found in the lesions/anatomic sites, with the presence of HPV malignant types (18, 31, 33, 35, 45, 51, 52, and 58), “probably carcinogenic” (HPVs 53 and 66) and non-carcinogenic (6, 11, 40, 42, 43, 44, and 54) (Herfs et al., 2012). Gurvich et al. (2004) study also postulated that the ectoendocervical squamocolumnar (SC) junction of the cervix cells could be the prime target for cervical carcinogenesis. The authors observed higher immunohistochemistry biomarker levels [keratin (Krt) 7, anterior gradient (AGR) 2, cluster differentiation (CD) 63, matrix metalloproteinase (MMP) 7, and guanine deaminase (GDA)]. Those biomarkers possessed a specific labeling in the cuboidal cells at the interface of the transformation zone of the squamous cervical epithelium and the endocervix. They found, in the totality of lesions, in junctional cervical intraepithelial neoplasia (CIN1 and CIN2/3), squamous cell carcinoma (SCC), adenocarcinoma *in situ*, or invasive adenocarcinoma (Herfs et al., 2012). In addition, HPV E6 and E7 gene expression and gene function were different in distinct epithelial sites, such as the endocervix, the cervical transformation zone, and the ectocervix (reviewed by Egawa et al., 2015).

**Figure 1:**
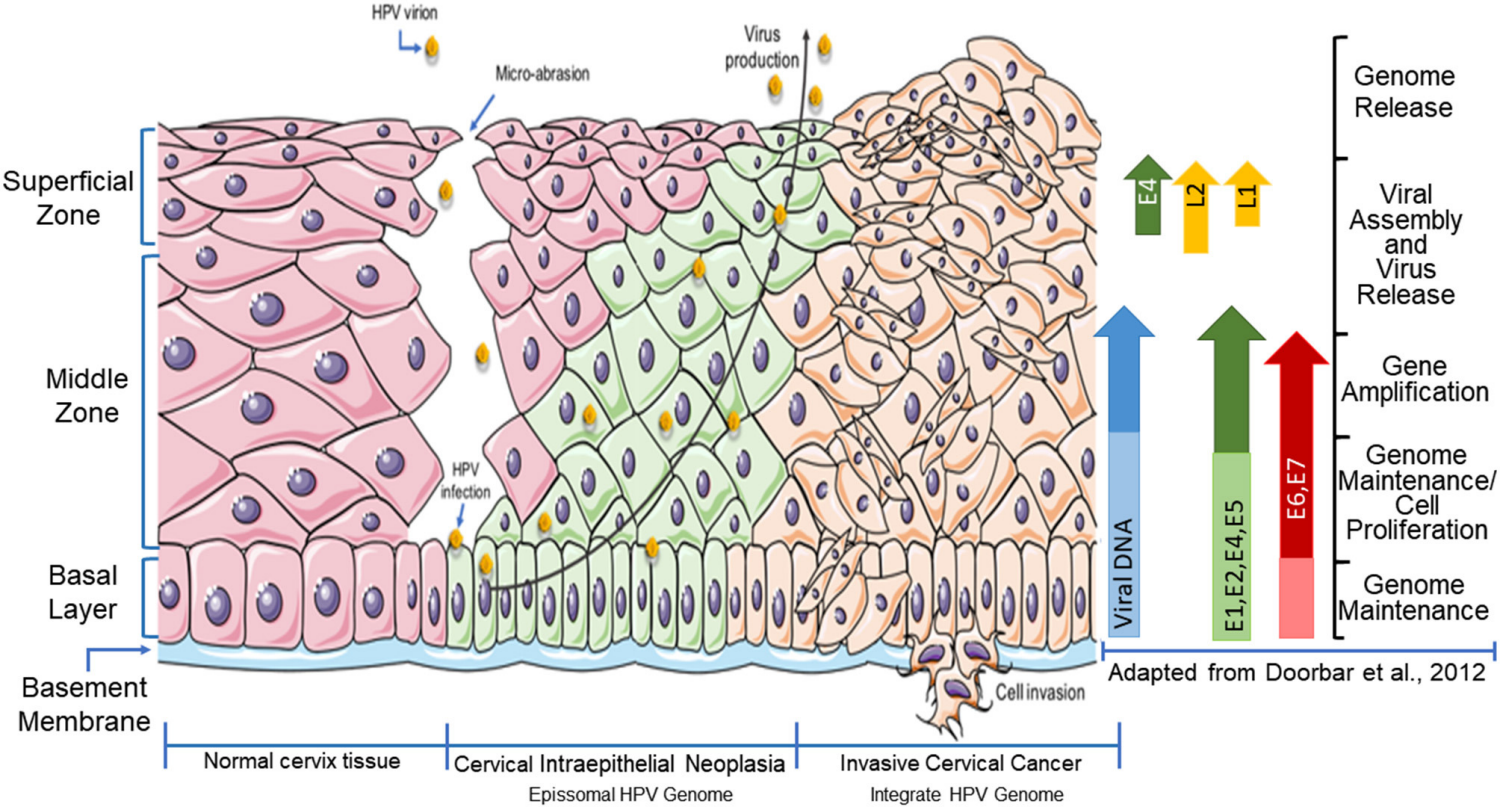
Schematic representation of high-risk HPV life cycle infection and tumor progression in the cervical tissue. Viral DNA load; *E1, E2, E4, E5, E6*, and *E7* expression; and *L1* and *L2* expression were demonstrated through epithelium layers (adapted from Doorbar et al., 2012). HPV, human papillomavirus; early genes (*E1, E2, E4, E5, E6*, and *E7*) and late genes (*L1* and *L2*).

Then, the protein E2 recruits E1 in order to increase the number of copies of viral episomes, which continue increasing upon epithelium differentiation (Figure 1). In cervical intra epithelial lesions (CIN1) with high-risk HPV infection (i.e., HPV16), the medium and upper differentiating epithelial layers, E4, E5, E6, and E7 proteins are highly expressed leading to uncontrolled cell proliferation, and when L1 and L2 proteins are expressed in the uppermost layer of the epithelium, the viral life cycle is completed (Doorbar et al., 2012). So, the viral genome is encapsulated and the release of mature virions occurs (Doorbar et al., 2012).

**Figure 2:**
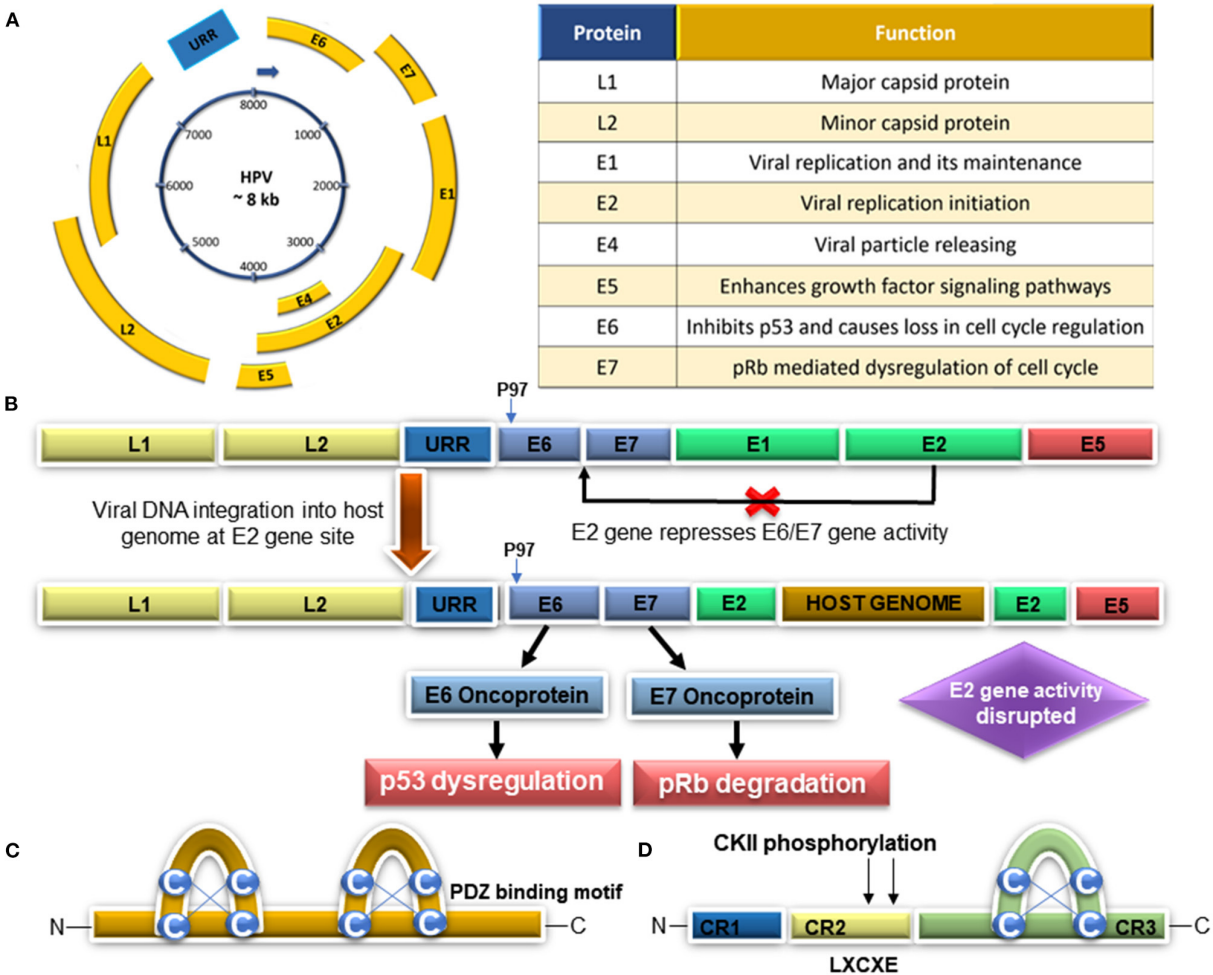
**(A)** Schematic HPV genome representation and viral protein functions. **(B)** Persistent infection with viral integration mechanism: abrogation of *E1* and *E2* gene repression and *E6* and *E7* upregulation, followed by p53 deregulation and pRb degradation. **(C)** PDZ protein binding is critical for E6 protein-transforming activities. **(D)** Representation of the *E7* gene structure and LXCXE-biding motif to pRb (Modified from Stanley, 2012; Adapted from Pal and Kundu, 2020).

Although HPV is a necessary agent associated with cervical carcinogenesis, it does not seem to be a sufficient cause. Therefore, some cofactors could be involved in the cervical cancer development, such as long-term use of hormonal contraceptives, high parity, tobacco smoking, and HPV coinfection with different microorganisms [HIV, chlamydia trachomatis (CT), and herpes simplex virus type-2 (HSV-2)] (reviewed by Muñoz et al., 2006; reviewed by Sanjosé et al., 2018). However, those cofactors could be related to higher HPV infection or increase its gene expression. The combination of sociodemographic status and multiple sexual partners are cofactors to higher prevalence of high-risk HPV infection in cervical cancer (Fernandes et al., 2009; Ardhaoui et al., 2016; Kasamatsu et al., 2018). The study in Costa Rica from the Schiffman team verified that HPV genotype coinfection was associated with an increased risk of cervical disease and that coinfecting genotypes lead to cervical disease (Chaturvedi et al., 2011). Animal studies indicate that sex steroid hormones (estrogen and endogenous progesterone) are capable of inducing cancer in *in vitro* and *in vivo* experiments associated with HPV E2, E6, and E7 proteins (reviewed by Hellberg, 2012). A recent study demonstrated an interplay between cigarette smoke exposure and HPV16, which resulted in EGFR-PI3K-AP-1 signaling that favors p97 activity and E6 and E7 overexpression in CaSki and SiHa cells (Muñoz et al., 2018). The persistent infection by high-risk HPV types, the E6 and E7 viral oncoproteins, is due to their impact on apoptosis/senescence and proliferation *via* inactivation of p53 and pRb, contributing to cervical epithelial transformation in cancer (reviewed by Ghittoni et al., 2015; reviewed by Hoppe-Seyler et al., 2018). In addition, E6 and E7 oncoproteins promote genetic instability of the host cell DNA by deregulating cellular factors involved in epigenetic reprogramming (reviewed by Hoppe-Seyler et al., 2018). Regarding HPV coinfection with other sexually transmitted microorganisms, CT infections favor the entry and persistence of multiple high-risk HPV types, which leads to viral integration, apoptosis inhibition, overexpression of *E6/E7* oncogenes, and cell transformation (Paba et al., 2008). In cultures derived from a transfection of 4 different HPV-immortalized keratinocyte cell lines (CX18-1, CX16-5, CX16-2) engineered with HSV-2 DNA, the transfection with HSV-2 DNA was capable of inducing the tumorigenic conversion of the HPV-immortalized human genital epithelial cells (Paolo et al., 1998). The HPV genome (Figure 2A) is constituted by a circular DNA double strand containing about 8,000 base pairs, encoding a total of eight genes, and is divided into three regions: the *early genes* (*E*), the *late genes* (*L*), and the *long control region* (*LCR*) (reviewed by Ghittoni et al., 2015). The gene expression of the cancer-associated high-risk HPV type is modulated by cis-regulatory elements located within *URR* (Hoppe-Seyler and Butz, 1993). The replication of HPV genomes requires E1 and E2 proteins as the viral trans-factors and the replication origin, located in the URR, as a cis element. Viral genes that are apparently expressed at low levels in these early phases of the replicating viral life cycle in the basal cells encompass the *E6* and *E7* genes as well as *E1* and *E2* (Figure 2B), which are all controlled by the *P* early promoter (*P97* in case of HPV 16 and 31, *P107* in case of HPV 18), which is located upstream of these genes in the viral *Upstream Regulatory Region* (*URR)* (reviewed by Doeberitz and Vinokurova, 2009).

HPV integration (Figure 2B) and gene replication are a pivotal machinery to cancer, triggering uncontrolled cellular proliferation, angiogenesis, invasion, metastasis, and unrestricted telomerase activity along with the evasion of apoptosis and growth suppressors' activity. The *E1* and *E2* genes are necessary for the maintenance of the viral genome in the host cells since they are responsible for the initiation of replication and the recruitment of the cellular DNA polymerase necessary for this process. The expression of the viral *E2* transcriptional repressor is generally lost upon viral genome integration resulting in dysregulated viral gene expression from the viral *LCR* or *URR*. HPV *E6* and *E7* genes are consistently expressed even after genome integration, and the expression of these proteins is necessary for its maintenance. The continued expression of the E1 and E2 replication proteins from integrated genomes causes focal genomic instability at the integration locus. The integration event disrupts the *E2* gene, alleviating *E2* transcriptional repression of the *E6* and *E7* genes (McBride and Warburton, 2017). Therefore, the *E6* and *E7* gene products proved to be the main responsible for the cell transformation process. It has been proposed that the HPV integration into the host genome occurs after a break in the *E2* gene, which has been described as the main repressor of *E6* and *E7* oncogene expression (Figure 2B). E6 proteins bound to LxxLL peptides from the ubiquitin ligase E6AP. E6 zinc domains and a linker helix form a basic–hydrophobic pocket, capturing helical LxxLL motifs, which could interact with different peptides. This interaction allows different E6 proteins to capture different panels of host proteins (Zanier et al., 2013) Mutational inactivation of the LxxLL-binding pocket disrupts the oncogenic activities of both E6 proteins (Zanier et al., 2013). Moreover, E6, the LxxLL motif of E6AP, and the core domain of p53 render the conformation of E6 competent for interaction with p53 by structuring a p53-binding cleft on E6. The mutagenesis of critical positions at the E6–p53 interface disrupts p53 degradation (Martinez-Zapien et al., 2016). E7 is a phosphoprotein with ~100 amino acid residues and three conserved regions, CR1, CR2, and CR3. Conserved region 2 contains the LXCXE (Leu-X-Cys-X-Glu) motif (Figure 2C), which is essential in the association with its targets, and CR3 forms a zinc finger structure (McLaughlin-Drubin et al., 2012). P130 and p107 share a distinct pocket domain necessary for binding E2F transcription factors and LXCXE motif-containing cellular proteins, including the D-type cyclins and histone deacetylases (HDACs). In their hypo- or unphosphorylated forms, the pocket proteins negatively regulate cell cycle progression through interaction with E2F/DP heterodimers and the recruitment of HDACs that promote chromatin condensation and repress transcription (Baker et al., 2005). The E7 oncoprotein interacts with p105, p107, and p130 proteins through the same motif (LXCXE) that E7 interacts with pRb. Therefore, p130 and its related proteins, pRB and p107, are important regulators of cell cycle progression, senescence, development, and differentiation (Nurshamimi et al., 2016; reviewed by Gupta and Mania-Pramanik, 2019). Moreover, the CR2 region of E7 contains the CKII phosphorylation site and the LXCXE-binding motif involved in binding to proteins such as the retinoblastoma tumor suppressor—pRb (reviewed by Gupta and Mania-Pramanik, 2019). HPV E7 proteins, which differ markedly in their ability to interact directly with pocket proteins, can both cause p130 degradation and lead to p130-DREAM complex disruption, resulting in the promotion of cell proliferation (Nurshamimi et al., 2016).

**Figure 3:**
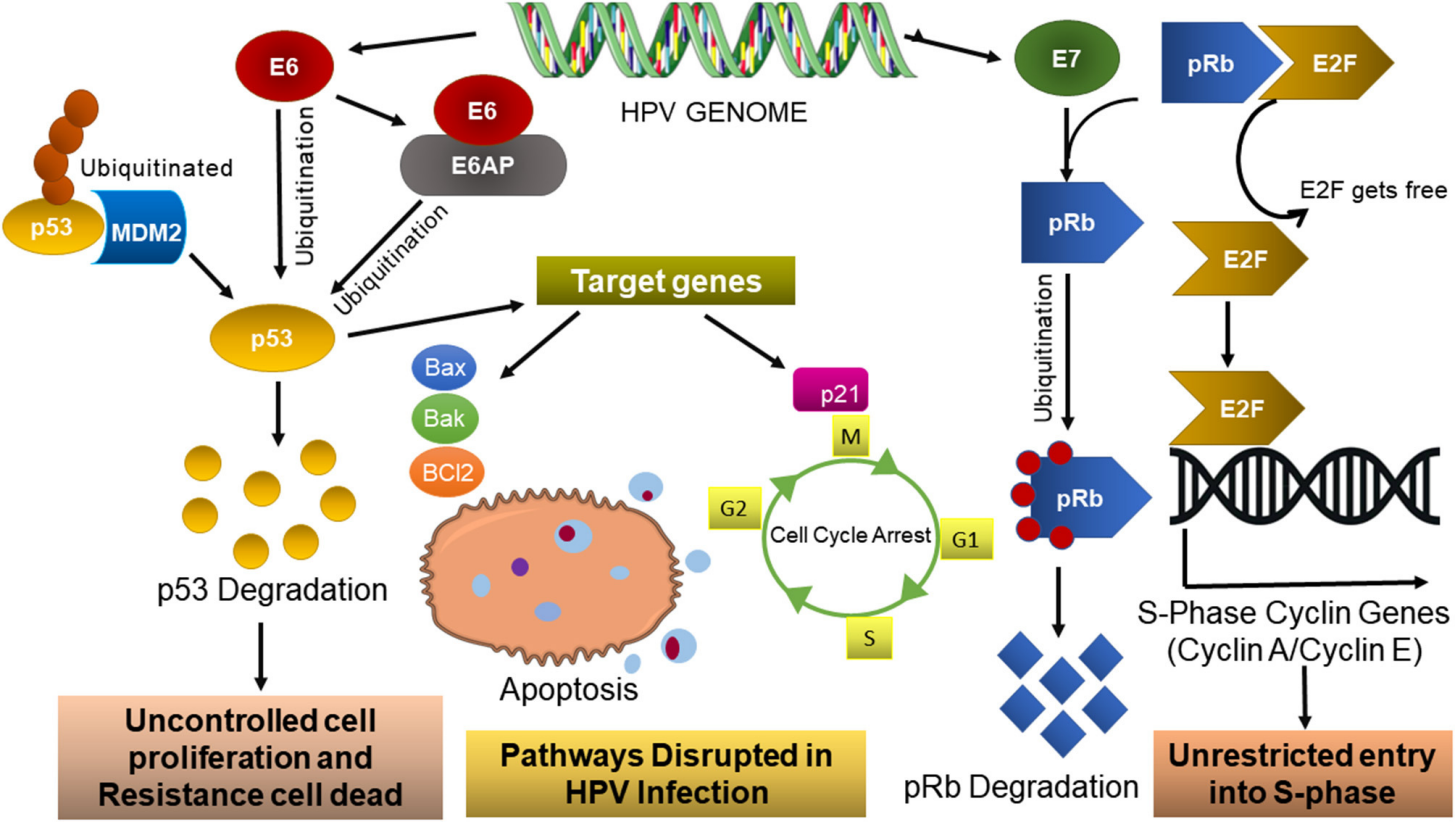
HPV dysregulation pathways. HPV persistent infections result in E6/EAP and E7 oncoprotein and its association with the main routes to cellular transformation (adapted from Pal and Kundu, 2020).

It is well-established that the carcinogenesis associated with HPV mainly depends on viral early genes, resulting in cellular transformation. The *E6* and *E7* oncogene upregulation is pivotal to tumorigenesis and indirectly influences cell pathway dysregulation, such as apoptosis, proliferation, growth, and motility. Particularly for cervical cancer, E6 interacts with the LXLL motif of E6-associated protein (E6AP), an E3 ubiquitin ligase that works as a connecting bridge between E6 and p53 (Figure 3), inducing p53 degradation through the proteasome pathway and consequently blocking the p53-dependent apoptosis Bcl2 family. High-risk alpha HPV E7 proteins target the retinoblastoma tumor suppressor pRB for proteasomal degradation. This causes aberrant, persistent S-phase entry, thereby sustaining proliferative signaling (reviewed by Mesri et al., 2014).

In persistent infection, the accumulation of DNA damage due to interactions of high-risk HPV E6 and E7 with p53 and pRb causes apoptosis inhibition and uncontrolled proliferation, which after a long time may lead to the alteration of chronically infected cells into cancer cells (Hoppe-Seyler et al., 2018).

The HPV E6/E6AP (Figure 3) oncoprotein interacts with several host cell proteins and thus presents different functions, such as intrinsic apoptosis pathways (downregulation of Bcl2 anti-apoptotic protein as well as upregulation of Bak and Bax pro-apoptotic protein), downregulation of p53 protein, and dysregulation of the cell cycle (downregulation of P300/CBP complex protein) (reviewed by Patel et al., 1999 reviewed by Estêvão et al., 2019). Consequently, the p53 levels are low and cyclin-dependent kinases (CDKs) are active, stimulating the cell cycle progression in an uncontrolled way and causing the accumulation of genetic mutations (reviewed by Estêvão et al., 2019). The E6 oncoprotein is found in the nucleus and interacts with several targets, presenting diverse functions, like deregulation of cell cycle (pRB protein and p107/p130 downregulation) (Estêvão et al., 2019). In high-risk HPV infections, the oncoprotein E7 alters this regulatory mechanism through its binding to the pRb protein (Figure 2). HPV-type 16 E7 also targets the pRb-related proteins p107 and p130 for destabilization by a proteasome-dependent mechanism. pRb degradation, not solely binding, is important for the E7-induced inactivation of pRb (Gonzalez et al., 2001). This way, pRb loses its ability to bind E2F transcription factors (Figure 3), and the release of these factors will stimulate the transcription of E2F-dependent genes necessary for DNA replication, resulting in cell cycle progression. E7 high-risk HPV contributes to this disruption cellular event, abrogating the inhibitory activities of the cyclin-dependent kinase inhibitors p21 and p27 (reviewed by Estêvão et al., 2019; Pal and Kundu, 2020). So, this ensures that the cell remains in an S-phase-competent state, which is vital for the viral life cycle.

Acetylation and deacetylation could represent different *E6* and *E7* oncogene expressions, and their activity is the hallmark of cancer cells associated with HPV infections. The first approach was the comprehensive review performed by Munger's team (Soto et al., 2017). HPV E6 and E7 oncoproteins can associate with HATs and modulate its activity. HPV E6 inhibits acetylation of p53, while HPV E7 forms a protein complex, acetylating pRb and abrogating HAT activity. Moreover, the HPV E7 oncoprotein interacts with class I HDACs, promoting reversal of acetyl modifications on histone lysine residues. E7 is associated with HDAC1/2, which occurs in a dependence on *RB*-independent manner and histone deacetylation complex by which remodeling chromatin structure happens through the deacetylation of histones. The association of E7 and HDAC1/2 does not result in the inhibition of HDAC activity but does play a role in HPV E7-associated transcriptional regulation. This association increased E2F2-mediated transcription levels in differentiating cells, affecting S-phase progression (Soto et al., 2017). *In vitro* experiments with deacetylase sirtuin 1 (HDAC III deacetylase family) and HPV31 have demonstrated that E6 and E7 acted synergistically, increasing SIRT1 levels through a posttranscriptional mechanism. In addition, E6 binding to p53 contributes to SIRT1 protein overexpression, as does E7 binding to both retinoblastoma protein (Rb) and histone deacetylases (HDACs). This study concluded that SIRT1 regulates HPV viral DNA basal replication and amplification and modifies histones bound to HPV genomes (Langsfeld et al., 2015). Tip60 is histone acetyltransferase responsible for acetylating the ε-amino groups of lysine residues on target proteins, including p53, DNA repair proteins, and histone H2A. Activated Tip60 acetylates (Ac) the ataxia–telangiectasia mutated (ATM) kinase, resulting in ATM autophosphorylation. The activated ATM then induces the phosphorylation (P) signaling proteins and γ-H2AX, which contribute to HPV genome amplification (Hong et al., 2015). The association of E7 and HDAC1/2 plays a role in HPV E7-associated transcriptional regulation suppression of interferon response factor 1 (IRF1) and blockage of HIF-1α (hypoxia-inducible factor-1), triggering pro-angiogenic factors by HPVE7 (reviewed by Gupta and Mania-Pramanik, 2019).

### From Histone to Enzymatic Acetylation/Deacetylation: The History

The studies of free histones were started by Professor Kossel, the founder of modern biochemistry, who separated the nucleic acids in sediments and histones in the supernatant (Mirsky and Ris, 1951). Professor Albrecht Karl Ludwig Martin Leonhard Kossel was a professor of physiology at the Heidelberg Kossel University and Study of Proteins and was a Nobel Prize winner, known for his elucidation of the chemistry of the nucleic acids and nuclear chromatin proteins (Mathews, 1927). His research on the nucleus mentioned a protein isolated from the red blood corpuscles of birds, which was different from Miescher's protamine (isolated before from the nucleus of salmon sperm), and Kossel named it histone (Kossel, 1884). Histone was described as a combination of nucleic acid with proteins, which Kossel called nucleoprotein, also finding basic amino acids in calf-thymus' histones (Kossel, 1928). The protamine was composed of small, arginine-rich residues, observed in the nuclear proteins of sperm cells in the haploid phase of spermatogenesis, and is essential for sperm DNA condensation and stabilization. In contrast, Kossel verified a basic amino acid composition in the histone (Mathews, 1927; Daly et al., 1950). The histones' characteristics, noted before Kossel's work, possessed high arginine content, which depended on how the histone had been chemically precipitated (cited by Daly and Mirsky, 1955), and several studies examined histone amino acid composition (Luck et al., 1958; Phillips, 1958; Phillips and Johns, 1965). In concomitant investigations, other studies were trying to better characterize histones' amino acid content, which on calf-thymus histones the chief presence of proline, alanine, and lysine in the N-terminal position was verified (Luck et al., 1958; Phillips, 1958). The N-terminal alanine, lysine, and glycine residues in the histones varied according to the different histone chemical extraction methods (Phillips, 1958; Phillips and Johns, 1959). At the beginning, the calf-thymus' histones had proline end groups and were associated with the slightly lysine-rich part (Phillips, 1958). Through the biochemistry procedures for the calf-thymus' histone extraction, histone's amino acids were extracted more easily with lysine-rich residues (Daly and Mirsky, 1955; Luck et al., 1958) and were confirmed in both calf thymus and wheat-germ histone (Johns and Butler, 1962). Further, chromatin was described as a nucleosome, composed by an octamer of histones H2a, H2b, H3, and H4, with about 200 base pairs of DNA involving these histones, which made the chromatin fiber flexible (Kornberg, 1974; Noll, 1974; Thomas and Kornberg, 1975). Histone H1 was considered a variable linker region, which stabilizes the interaction of adjacent nucleosomes (Noll and Kornberg, 1977). Through crystallography assays, this histone was characterized as a core of a pair of histone types H2A, H2B, H3, and H4 with a supercoiling of DNA around the core (Dubochet and Noll, 1978).

The chromatin was described as an RNA–DNA–protein complex, protecting new RNA against RNAse activity in the chromatin from pea embryos (Bonner et al., 1961). It became known that DNA was present in at least two forms, namely, DNA itself and DNA bound in the nucleus–histone complex (Huang and Bonner, 1962). Then, the histones could inhibit RNA synthesis in the nucleus (Allfrey et al., 1963), and the selective remotion of the histones led to increased rates of messenger RNA synthesis (Allfrey et al., 1963). Furthermore, the RNA-synthesis inhibition was associated with the lysine-rich histone fractions or a histone complex, which favored DNA application as a primer for RNA synthesis (Allfrey et al., 1963). So far, a precocious relationship between histone-rich domains in lysine, histone modification, and RNA synthesis has been found (Allfrey et al., 1964). Afterward, the histones could be further modified by the attachment of acetyl, methyl, or phosphoryl groups in the preformed lysine peptide chains. The acetyl group has been found at the amino group terminal in the polypeptide chain attached at the ε-amino group of lysine residues (Phillips, 1963; Phillips and Johns, 1965). Early studies on calf thymus nuclei also verified an association between acetylated histones on RNA polymerase reactions and the histones' modifications by acetylation and that DNA-histone binding could influence the rate of RNA synthesis (Huang and Bonner, 1962; Allfrey et al., 1964). Moreover, the chromatin DNA was available for transcription by RNA polymerase, which remained inert as it was physically repressed by histones, and when these were removed from chromosomal DNA, the genetic material was derepressed (Bonner, 1965). The same conclusion was reached in human lymphocytes, when the histone acetylation was also followed by reversible changes of DNA attached to the histone's and also modifying RNA polymerase activity and RNA synthesis (Pogo et al., 1966). Seeking further scientific evidence, Pogo et al. (1966) and Allfrey et al. (1964) verified a high turnover of this N-terminal acetyl group in histones and increased RNA synthesis in metabolically active tissues (i.e., spleen, liver, thymus, hepatocytes, and tumor cells) (Byvoet, 1968; Pogo et al., 1968). This made researchers aware of the enzymatic modification of histones. Histones isolated from rat's liver nuclei and chicken reticulocytes demonstrated that the histone modifications were promoted through the transfer of acetate from coenzyme A by an acetokinase (Marchis-Mouren and Lipmann, 1965; Gallwitz, 1968). Nohara et al. (1968) concluded an enzymatic acetylation of histones in a stable lysine-rich chain, which was confirmed in adrenal tumor cells (Jungmann et al., 1970). The presence of histone acetyltransferase in the chromatin isolated from rat's isolated nuclei was also demonstrated (Racey and Byvoet, 1971). Thereafter, different histone acetyltransferases were found in rat thymus' nuclei or rat's hepatoma cell lines, promoting acetylation in histone f2a_1_ instead of histone f3 (Gallwitz and Sures, 1972) or in histone H4 instead of H3 (Garcea and Alberts, 1980). The N-terminal acetylation of this histone decreases the interaction with DNA in histone H4 lysine residues and promotes accessibility to RNA synthesis (Horiuchi et al., 1978a,b). Furthermore, the cellular activity was observed in different cells and at different phases of the cell cycle depending on the acetylation status of chromatin on histone side chains (Marushige, 1976). In addition, the highly metabolically active histone acetylation occurred in normal and tumorous cells (Moore et al., 1979). Those past studies brought the basic knowledge that histone enzymatic acetylation levels at lysine-rich chains were associated with high levels of cellular RNA transcription in proliferative and cancer cells.

**Figure 4:**
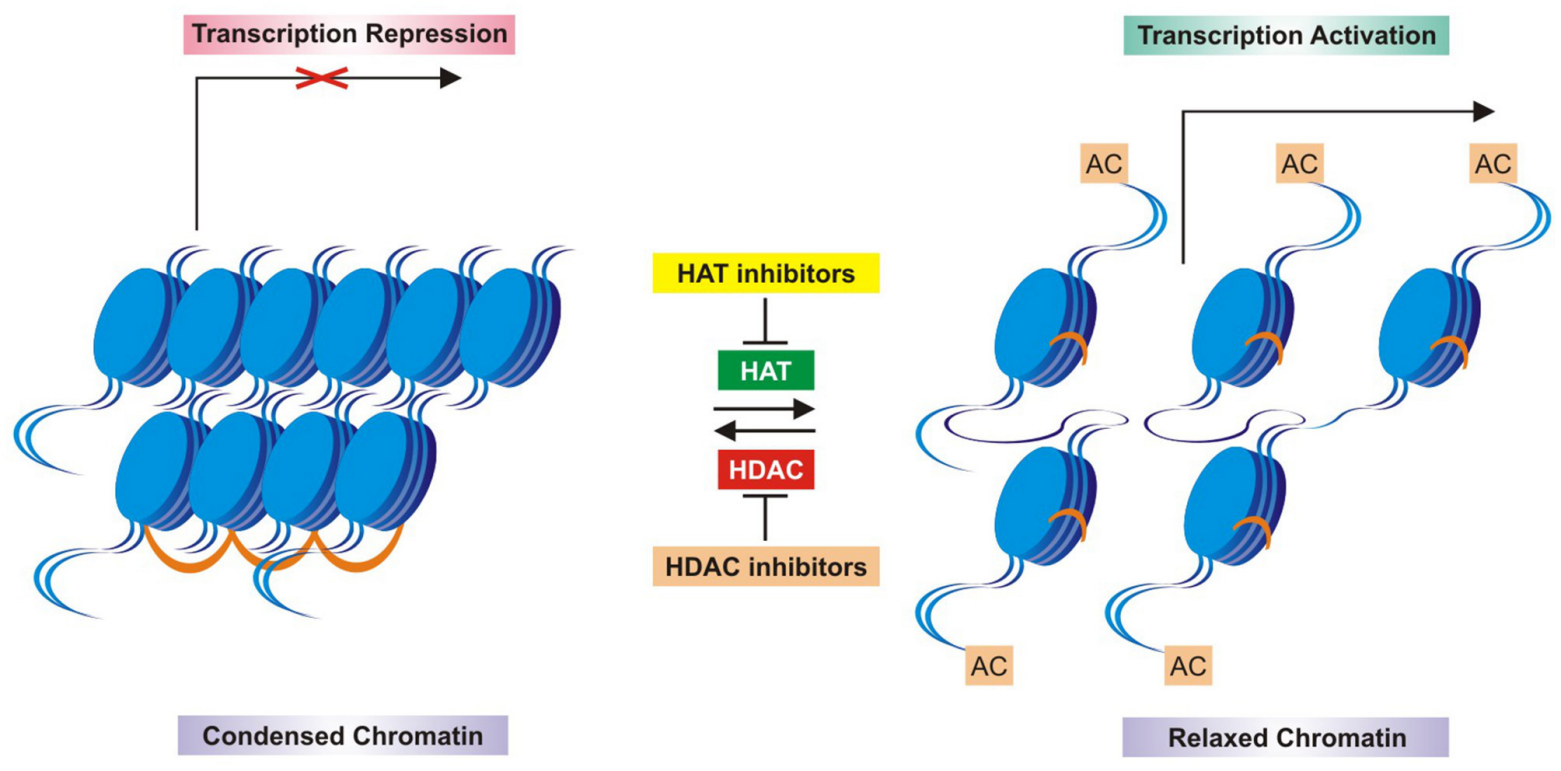
Schematic mechanism of histone acetylation and deacetylation enzymatic control and inhibitors. HAT, histone acetyl transferase; HDAC, histone deacetyl transferase; HDACi, HDAC inhibitors.

In concomitant studies, the enzymatic deacetylation of histone in calf thymus demonstrated slight modifications of the histone structure by acetylation and deacetylation, reflecting on the way to modify the chromatin and RNA synthesis (Allfrey et al., 1964; Inoue and Fujimoto, 1969). Then histone acetylase and deacetylase enzymes became important mechanisms for genetic control in higher organisms (Inoue and Fujimoto, 1969), and the acetyl turnover by deacetylase activity happened in active metabolic cells and tumors (Libby, 1970; Horiuchi and Fujimoto, 1972; Fujimoto and Segawa, 1973; Reeves and Candido, 1980). Moreover, the histone deacetylase enzymatic activity occurred in both free histone and chromatin-bound histone complexes (Vidali et al., 1972; Kaneta and Fujimoto, 1974). In mammalian cells, a deacetylase was discovered, which supported the statement that histone deacetylase was a key regulator of eukaryotic transcription (Taunton et al., 1996). In summary, the acetylation and deacetylation of histones were enzymatic processes, allowing RNA polymerase to promote gene transcription or to return it to a repressed state (not transcriptional). This was considered a hallmark of epigenetics, especially in cancer (Horiuchi et al., 1981; Hull, 1982; Davie and Hendzel, 1994). An important concept arose on histones' modifications in terms of terminology: “writers,” “erasers,” and “readers.” The “writers” were defined as enzymes (HAT, histone methyltransferase or kinases), posttranslationally adding acetyl, methyl, or phosphoryl groups to histones; the “erasers” were enzymes (HDAC, demethylase or phosphatase) promoting opposite posttranslational histone modifications; the “readers” were regulatory proteins which recognized domains that complemented specific posttranslational modifications: bromodomains for acetylation, chromodomains for methylation, and 14-3-3 phosphobinding proteins for phosphorylation (Soto et al., 2017; Zhao et al., 2018). Several studies follow these past works, which greatly contributed to the current knowledge and epigenetic therapies against tumors based on the inhibition of histone enzymatic activity of deacetylation (Figure 4).

In humans, 18 isoforms of HDAC have been described and subdivided in four main classes (Johnstone, 2002; Fass et al., 2013). Class I is constituted by HDACs 1, 2, 3, and 8; class II is constituted by HDACs 4, 5, 6, 7, 9, and 10; class II includes SirT 1–7 and class IV presents HDAC11. The HDACs that belong to classes I, II, and IV are zinc-dependent enzymes, while the class III HDACs are NAD^+^ dependent (Bolden et al., 2006; Fass et al., 2013; Zhao et al., 2018).

### HDAC Inhibitors (HDACi) for Cervical Cancer and HPV

**Figure 5:**
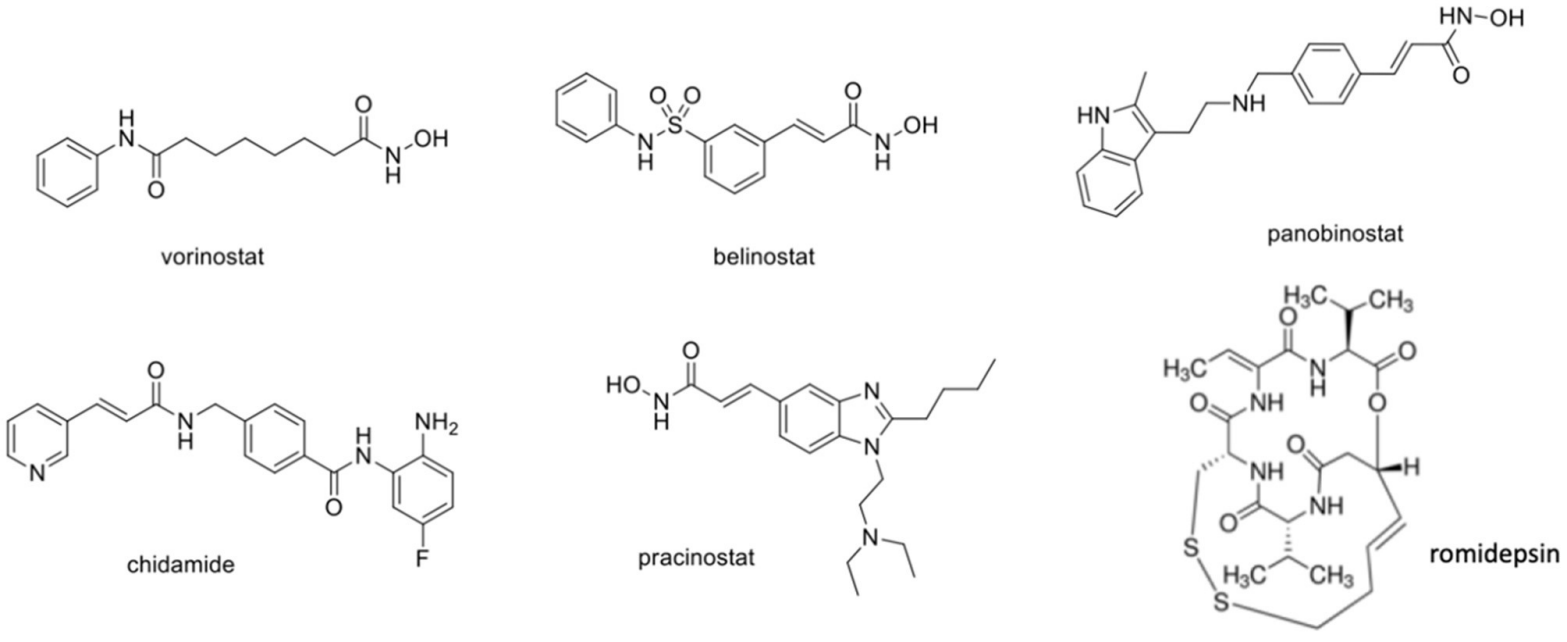
Chemical structure of FDA-approved HDACi. FDA, Food and Drug Administration; HDACi, histone deacetylase inhibitors.

Several works have been conducted using HDACi against several HPV cell lines, including HeLa (cervical adenocarcinoma with HPV18 10–50 number of viral copies), CasKi (squamous cervical cancer with HPV16 60–600 number of viral copies), or SiHa (squamous cervical cancer with HPV16 1–2 number of viral copies) (Meissner, 1999). Once HDACs are involved in the acetylation not only for the histone but also for non-histone proteins (as for example, p53), its inhibition can interfere in a series of biological pathways related to cellular growth, cellular signal transduction, and death (Gregoretti et al., 2004; Yang and Seto, 2008). Due to the overexpression of HDAC in many types of cancer, including cervical cancer, it is known that HDAC inhibitors act by reducing tumor development, being used alone or in combination with other drugs. After research efforts in the last years, only few HDACi drugs were approved by the FDA, including vorinostat (also known as SAHA) (2006), romidepsin (2009), belinostat (2014), panabinostat (2015), chidamide (2015), and pracinostat (2016) (Figure 5) (Sangwan et al., 2018; Banerjee et al., 2019). Despite the effectiveness of these drugs against relapsing multiple myeloma and cutaneous and peripheral T-cell lymphomas, some limitations are still observed during monotherapy for solid tumors (Mottamal et al., 2015; Bae et al., 2018). Phase II trials using HDACi against solid tumors revealed that only a few patients have reached complete remission. Moreover, serious adverse effects including anemia, leukopenia, thrombocytopenia, pulmonary embolism, and deep vein thrombosis were reported for these drugs, raising concerns regarding its therapeutic use (La Cruz-Hernandez et al., 2015). The reason for this limited efficacy against solid tumors remains obscure; however, a hypothesis came out from a study involving a breast cancer/triple-negative, which revealed that the activation of a leukemia factor receptor (LIFR) could be involved in the reduced activity of HDACi. Specifically, the recruitment of bet-bromodomain-4 (BRD4) which is associated with the upregulation of LIFR in tumors could activate the signaling pathway JAK-STAT3, leading to HDACi failure (Fedele et al., 2017). In spite of this limitation, a combination of drugs involving HDACi, BRD4, and/or JAK inhibitors seems to be a promising approach, mainly for solid tumors (Hosford and Miller, 2014). In this section, we will discuss the efficacy of HDACi against cervical cancer. We will present the most relevant preclinical studies with new compounds coming from organic synthesis and natural products. The main findings with FDA-approved drugs regarding clinical trials will be presented.

#### Compounds at the Preclinical Stage

Chemically, the HDAC inhibitors can be classified into 4 main classes: hydroxamates, 2-amino-benzamides, cyclic peptide, and aliphatic acids (Sangwan et al., 2018). For the hydroxamate class, whose main representative is vorinostat (i.e., SAHA) (Figure 5), a general chemical structural pattern consisting of three components was established, namely, (a) zinc-binding group, generally containing a chelating subunit represented by hydroxamic acid or 2-aminobenzamide subunits; (b) a linker region, used as a spacer, which is able to connect the zinc-binding group to cap a tunnel subunit; and (c) a cap subunit that allows interaction with amino acid residues located outside the active site (Ganai, 2018). Molecular modifications on the chemical structures in some of these three components can be explored to obtain selectivity among the different HDAC isoforms.

Based on the different structures of all four classes of enzymes (HDAC class I–IV), barriers regarding selectivity must be overcome to avoid off-target effects. It is well-established that a high percentage of identity is found among HDAC belonging to the same class. For example, in class I, the percentage of identity of HDAC1 compared to HDAC2, HDAC3, and HDAC8 is 85.1, 59.1, and 40.5, respectively. For HDAC class IIa, the percentage of identity of HDAC4 compared to HDAC5, HDAC7, and HDAC9 is 61.6, 53.4, and 59.7, respectively (Micco et al., 2013; Ganai, 2018). Thus, it is necessary to characterize which HDAC inhibition is more prone to contribute to an anticancer effect to circumvent possible adverse effects coming from an unspecific activity.

Aberrant expression among distinct HDAC is related to different types of cancer. For example, for gastric and prostate cancer the overexpression of HDAC1 was reported (Choi et al., 2001), while for hepatocellular carcinoma high levels of HDAC5 were found (Feng et al., 2014). HADC6 is overexpressed in oral squamous cell carcinoma, while the aberrant expression of HDAC7 is described for pancreatic cancer (Takumi et al., 2006; Ouaïssi et al., 2008). An aberrant expression of HDAC class I was reported in more than 75% of the types of cancer of human tissues and noncancerous epithelium, including, breast, colon, esophagus, lung, ovary, pancreas, prostate, stomach, and thyroid cancer (Nakagawa et al., 2007).

For cervical cancer, the role of which specific HDAC could be the main target is still uncertain. Overexpression of HDAC8 was reported in HeLa cells. Specifically, HDAC8 binds to and performs deacetylation of alpha-tubulin at the Lys40 (K40) position. The previous role of deacetylase attributed to HDAC6 seems to be shared by HDAC8, which suggests apparent functional redundancy (Hubbert et al., 2002). After knockdown of HDAC8 using siRNA experiments, an interference in the cell cycle was observed, as well as an alteration in cellular migration and morphology, which pointed out that HDAC8i could be a useful target in cervical cancer cells (Vanaja et al., 2018). No selective HDAC8 inhibitor has been approved as a drug, but a number of compounds described in the literature show promising activity against HDAC8; however, few of those compounds were assayed against cervical cancer, demanding additional efforts to comprehend the involvement of this isoform in cancer development using those cellular lineages (Banerjee et al., 2019).

**Figure 6:**
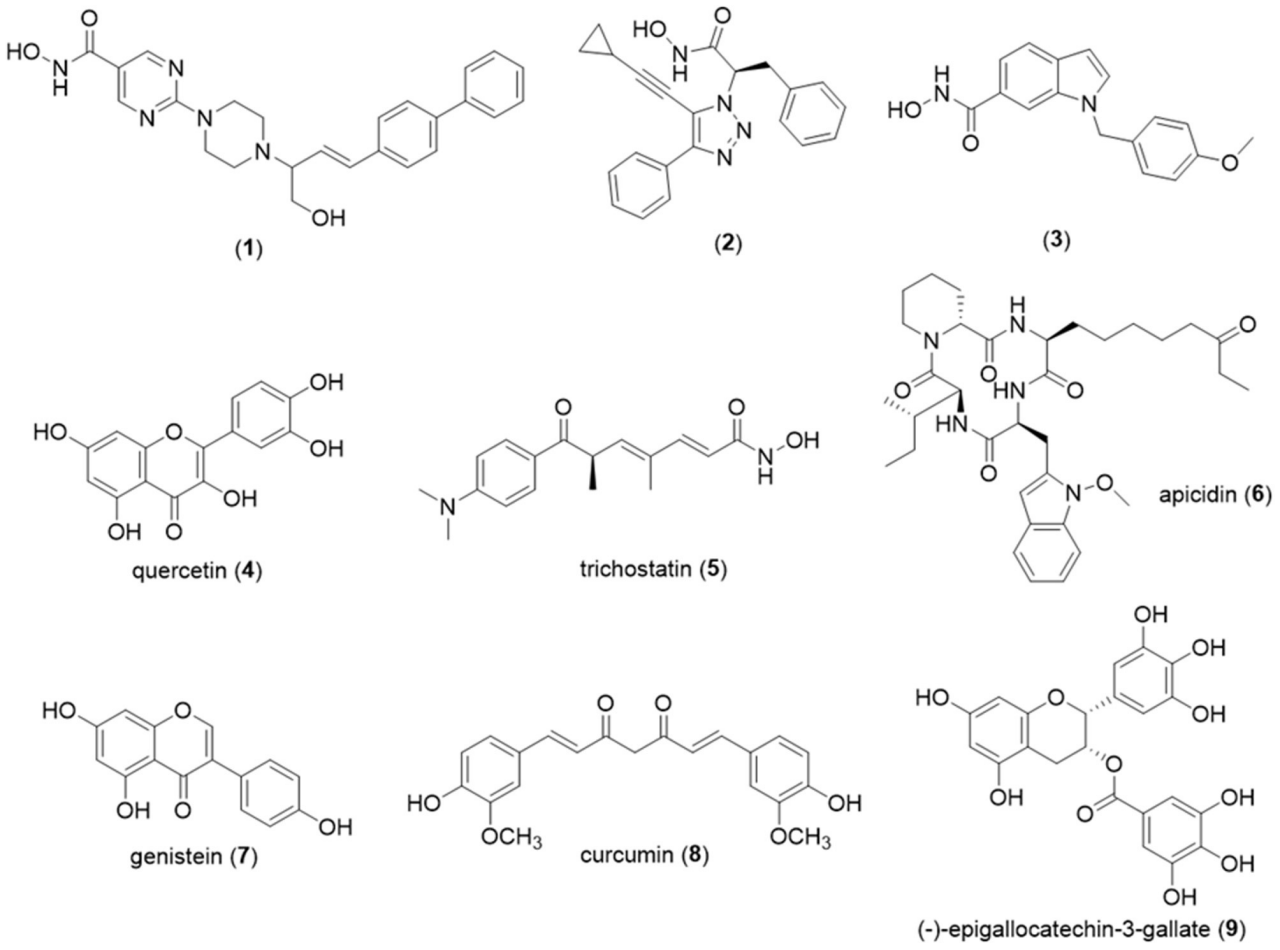
Chemical structures of HDAC inhibitors. HDAC, histone deacetylase.

Examples of potent HDAC8 inhibitors include 2-piperazinyl-5-pyrimidylhydroxamic acid derivatives. For this series, despite the absence of selectivity among the different isoforms in class I, compound (**1**) exhibited an IC_50_ value of 0.9 nM against HDAC8 and an antiproliferative effect against human ovarian cancer A2780 (IC_50_ = 29 nM) (Angibaud et al., 2010) (Figure 6). A selective HDAC8 inhibition was described for the phenyltriazole derivative (**2**) discovered after screening of an in-house large set of small molecules, followed by molecular optimization. For this most active compound (**2**), a IC_50_ value of 0.8 nM was found against HDAC8, while for HDAC-2, -3, -4, -5, -7, and -9 the value was superior to 20 μM. Thus, experimental data revealed for compound (**2**) more than 25,000 times selectivity for HDAC8 compared to those isoforms (Ingham et al., 2016). Another example of a selective HDAC8 inhibitor with an IC_50_ value equal to 10 nM previously described is compound (**3**) (Figure 6). This molecule (**3**) exhibited selectivity to HDAC8, once for the other class I isoforms; the IC_50_ values found were 4 μM (HDAC1), > 50 μM (HDAC2), >50 μM (HDAC3). In addition, *in vitro* assays showed that compound (**3**) was able to induce caspase-dependent apoptosis in cell lines derived from leukemias or T-cell lymphomas but failed to act against HeLa cells, since GI_50_ was superior to 20 μM (Balasubramanian et al., 2008).

Metalloproteinases (MMPs) are endopeptidases related to cellular behaviors such as cell proliferation, migration, adhesion, differentiation, angiogenesis, and apoptosis. MM2 and -9 are metalloproteinases that can regulate cell migration and invasion of cancer cells being overexpressed in many human tumors and are important prognostic factors in cervical cancer. It has been described that patients with lymph node metastasis have shown reduced expression of HDAC10 (class IIb) compared to those without metastatic prognosis in human cervical squamous cell carcinoma. It was demonstrated that HDAC10 binds to the promoter regions of MMP2 and -9, resulting in downregulation of their expression through deacetylation of histones H3 and H4 and a block of RNA polymerase II binding. A mutant HDAC10 without histone deacetylation effect did not exhibit any suppressive effect on MMP2 and MMP9 genes. The *in vivo* evaluation of metastasis in nude mice injected with HeLa cells in their footpad has demonstrated that HDAC10-overexpressing cells still have the ability to metastasize; however, the number of positive lymph nodes has decreased. These effects highlighted the contribution of HDAC10 to decrease metastasis in human cervical squamous cell carcinoma (Song et al., 2013).

Another HDAC that seems to be involved in cervical cancer is HDAC2, whose overexpression was reported by Huang's team (Huang et al., 2005). In this study, HDAC1 and HDAC2 were examined in more detail in some samples of cervical and colon cancer. The results suggested that HDAC2 is upregulated, more present, and stronger in polyps of these cancers with the dysplasia transition when compared to HDAC1. The expression of HDAC2 mRNA was regulated more than twice in 9/16 tumors. The study also shows that the knockdown of HDAC2 in cells causes an increase in apoptosis (possibly through an upregulation of p21Cip1/WAF1, but independent of p53), which is confirmed in the results of Zhu et al., where they demonstrate that HDAC2 overexpression works as a protection for cells against apoptosis, which can be important to facilitate the development of tumors.

#### HDACi From Natural Products and Synthetic Compounds Active Against Cervical Cancer

Despite the absence of information regarding selective inhibition of HDACi, several works describe the anticancer effect of natural products and synthetic compounds against cervical cancer. Many of those compounds are HDAC pan-inhibitors. This section will describe some of these molecules pointing out their promising activity against cervical cancer.

#### Natural Products and Synthetic Compounds

Throughout the human history, natural products have constituted an important source of treatment to several health afflictions. The chemical diversity of natural products allows the discovery of original molecules that can be used as complex in phytomedicines or isolated compounds. An exhaustive list of natural products is currently used as drugs for the treatment of several types of cancer, including vinca alkaloids (i.e., vinblastine, vincristine), taxanes (i.e., paclitaxel, docetaxel), podophyllotoxin (i.e., tenoposide, etoposide), and camptothecin (i.e., irinotecan, topotecan), among others (Choudhari et al., 2020). It has been estimated that natural products have contributed to around 50% of all approved drugs against cancer from 1940 to 2014 (Newman and Cragg, 2016).

Natural product analogs and synthetic compounds have also been discovered, designed, and evaluated as HDAC inhibitors, such as valproic acid, hydroxamic acids derivatives, and 2-aminobenzamide derivatives, among others. Herein we presented the synthetic compounds able to inhibit HDAC enzymes and present antiproliferative property activity against cervical cancer cell lines.

**Figure 7:**
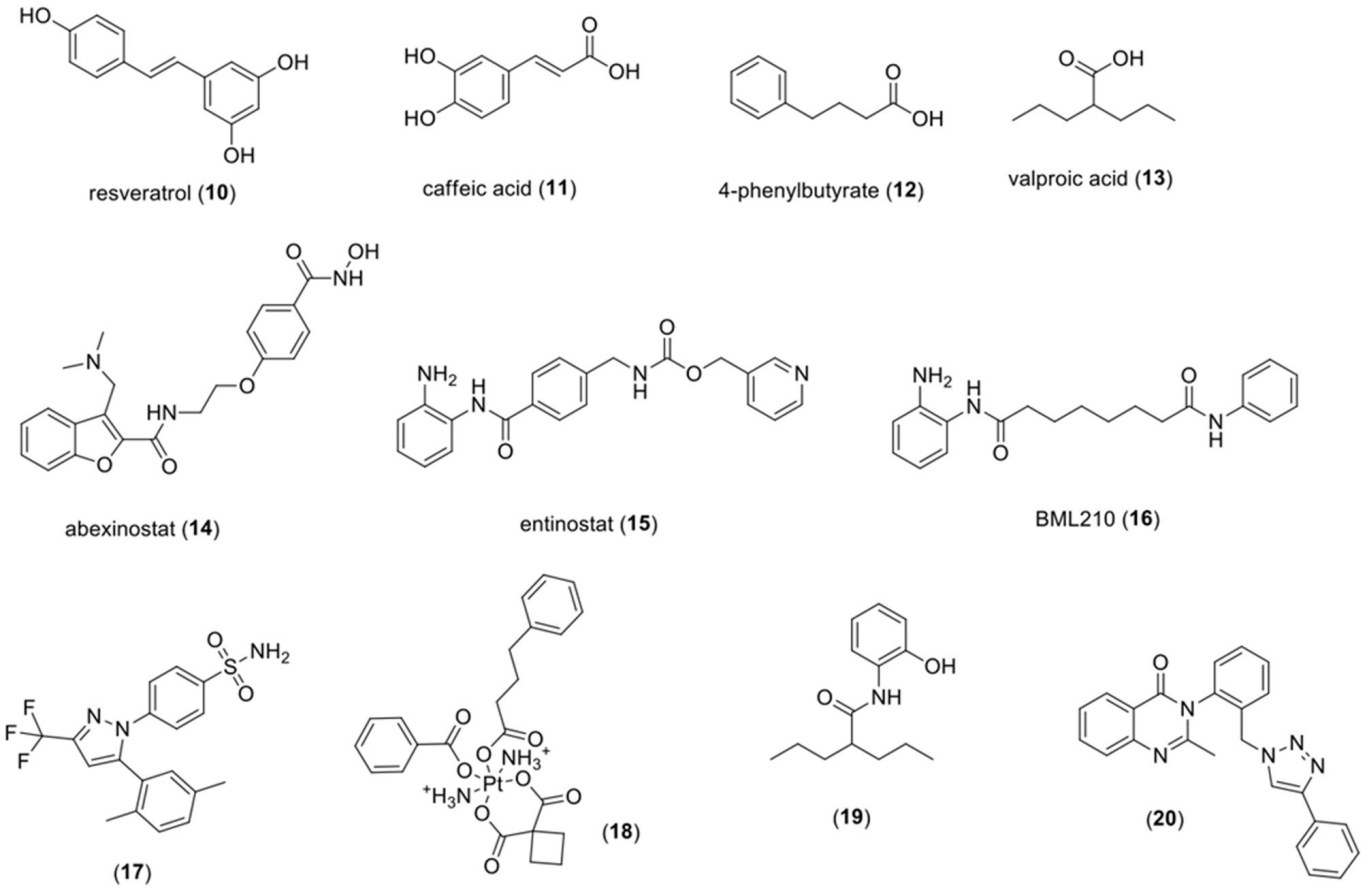
Chemical structures of HDAC inhibitors. HDAC, histone deacetylase.

Table 1 summarizes the natural and synthetic compounds, their proposed mechanism of action, general comments, and IC_50_ against HDAC enzymes. Figure 7 presents the chemical structures of compounds (**10**)–(**20**).

**Table 1:** HDAC inhibitors and mechanisms.

**HDAC inhibitors**	**Mechanisms/properties**	**General comments**	**References**	**IC_**50**_**
**Natural products**				
Quercetin **(4)**	HDAC2, HDAC4, HDAC7 and HDAC8	Induction of apoptosis by inhibiting epigenetic enzymes and reduction in DNA methylation. However, pharmacokinetic limitations and low solubility are still barriers to be overcome.	(Biswas et al., 2018; Sundaram et al., 2019)	HDAC1:26.72 μM HDAC8:15.4 μM HDAC6:43.39 μM
Trichostatin **(5)**	HDACs (class I and II)	Inhibition of human cervical adenocarcinoma HeLa cell growth in a dose- and time-dependent manner exhibiting IC_50_ value of 20 nM at 72 h.	(Noriyuki et al., 2007; Lauffer et al., 2013; You and Woo, 2014)	HDAC1:0.0049 μM HDAC2:0.0123 μM HDAC3:0.00141 μM HDAC8:0.213 μM HDAC4:2.4 μM HDAC5:0.871 μM HDAC7:0.663 μM HDAC9:3.7 μM HDAC6:0.000721 μM HDAC10:0.0116 μM
Apicidin **(6)**	Hyperacetylation of histones in cyclin E	Reduction of HPV16-E6 and HPV16E in transcript and protein levels, with decreased stability, showing the potential of this HDACi to manage tumor differentiation. Positively regulating hypoxia-induced prolyl factor 4-hydroxylase (PHD2) which is correlated with increased angiogenesis and tumor growth.	(Łuczak and Jagodzinski, 2008; Durczak and Jagodzinski, 2010; Huber et al., 2011)	HDAC1:0.3 μM HDAC2:1.2 μM HDAC3:0.98 μM HDAC8:0.26 μM HDAC4:>50.0 μM HDAC5:>50.0 μM HDAC6:>50.0 μM HDAC7:>50.0 μM HDAC9:>50.0 μM
Genistein **(7)**	Inhibitor of tyrosine kinase (PTK)	It acts against EGFR autophosphorylation, src kinases, and abl kinase. It is an HDAC6 inhibitor, which is later responsible for interfering with the HDAC6-HSP90 co-chaperone function involved in stabilizing androgen receptor protein.	(Akiyama and Ogawara, 1991; Basak et al., 2008)	
Curcumin **(8)**	HDAC pan-inhibitor	HDAC inhibition in non-resistant and resistant cervical cancer lines. The same effect was also observed for nonresistant SiHa cells and cisplatin resistant SiHa cells. Both cells exhibit high expression of HDAC1 and HDAC2, whose activity is decreased by pretreatment with this compound.	(Sarkar et al., 2014; Nelson et al., 2017)	HDAC8:115 uM
(-)-Epigallocatechin-3-gallate (EGCG) **(9)**	Ability to interfere with epigenetic mechanisms	The antiproliferative and apoptotic effect of EGCG (9) in other types of cancer cells are more described than in cervical cancer. The association of EGCG (9) with the synthetic retinoid compound Am80 leads to apoptosis due to the reduction of HDAC4, 5, and 6, and alteration of the acetylation levels of non-histone proteins, such as p53.	(Thakur et al., 2012; Fujiki et al., 2015)	
Resveratrol **(10)**	Modulation of transcription factors, cell cycle regulatory proteins, inhibition of angiogenesis and kinase proteins	WST-1 analysis in review showed that RVT (10) was able to reduce the viability of HPV + HeLa cells in a dose-dependent manner. In HeLa cells, RVT (10) at 50 μM downregulates the viral oncoprotein E6 and upregulates caspase 3. *In vivo* studies revealed both proteins E6 and VEGF were downregulated by this compound.	(Pavan et al., 2016; Chatterjee et al., 2018)	
Caffeic acid **(11)**	HDAC inhibition in nuclear extracts of HeLa cells	This phenolic compound was able to retard cell growth after 72 h of HeLa and SiHa cells. Molecular modeling studies suggest that caffeic acid (11) can inhibit HDAC2, which could be related to induction of caspase-3 mediated apoptosis and cell arrest in S and G2/M phases	(Bora-Tatar et al., 2009; Anantharaju et al., 2017)	HDAC−2,54 mM
**Synthetic compounds**				
Butyrates (**12**)	p21 induction, CDK2 inhibition and E2F transcription factor culminating in the dephosphorylation of pRb.	Butyrates are able to arrest G0/G1 cycle cells in both HPV type 16(+) SKGIIIa and Siha and HPV type 18(+) HeLa cells. However, it's inappropriate pharmacokinetics (i.e., short half-life, extensive, and fast metabolism) and low potency as HDACi limits its clinical use.	(Lea et al., 2004; Noriyuki et al., 2007,?)	HDAC−0.62 mM
Valproic acid (**13**)	HDAC pan-inhibitor; Activation of caspase-3, -8, and -9, increasing cleavage of PARP and alteration of mitochondrial membrane potential	Valproic acid exhibits ED_50_ values ranging from 0.32 to 0.78 mM against CaSki, ME180, and SiHa cells. It was also able to reduce up to 30% of tumor growth in an i*n vivo* tumor xenograft model using athymic mice implanted with HeLa cells. In a phase II study, VPA administration followed by epirubicin was well-tolerated.	(Gurvich et al., 2004; Chavez-Blanco et al., 2006; Münster et al., 2007; Noriyuki et al., 2007,?; Siraj et al., 2008; Han et al., 2013)	HDAC1:0.7 mM HDAC2:0.8 mM HDAC3:1 mM HDAC4:1.5 mM HDAC5:1 mM HDAC6:>20 mM HDAC7:1.3 mM HDAC10:>20 mM
**Hydroxamic acid derivatives**				
Abexinostat (**14**)	Increasing levels of acetylated histone H3 and phosphorylation of gamma H2AX in SiHa cells.	Abexinostat was active against several types of cancer. Treatment with abexinostat resulted in 80% of cell apoptosis and radio sensitizing property.	(Buggy et al., 2006; Banuelos et al., 2007; Evens et al., 2016)	Ki (μmol/L) HDAC1:0.007 HDAC2:0.019 HDAC3:0.0082 HDAC6:0.017 HDAC8:0.28 HDAC10:0.024
**2-Aminobenzamide derivatives**				
Entinostat (**15**)	Inhibition of HDACs 1, 2, 3, and 9	Entinostat presented anti-proliferative activity *in vitro* and *in vivo* against several cancer lines, including cervical cancer and a panel of gynecologic cell lines.	(Saito et al., 1999; Lauffer et al., 2013; Gorshkov et al., 2019)	HDAC1:0.2 μM HDAC2:0.5 μM HDAC3:0.3 μM HDAC4:>10 μM HDAC5:>10 μM HDAC6:5.9 μM HDAC7:>10 μM HDAC8:>10 μM HDAC9:>10μM HDAC10:>10 μM
N-(2-Aminophenyl)-N' phenyloctanol diamine (BML210) (**16**)	Reduction of HDAC1-5 and 7 levels in HeLa cells. Downregulation of DAPC genes	HeLa cells treated with BML210 (**16**) exhibited a high proportion of cells in G0/G1 cell cycle phase and accumulation in subG1. An association of retinoic acid (RA) and BML210 (**16**) induced apoptosis in a time-dependent manner, increased the levels of p21 and caused phosphorylation of p38 MAPK.	(Borutinskaite et al., 2006)	
**Miscellaneous**				
Dimethylcelecoxib (**17**)	Enhancer of HDAC1 activity	Compound (**17**) was able to downregulate EGR1 gene expression and upregulate NF-κB in HeLa cells. It improved the formation of complexes containing NF-κB and HDAC1, allowing its binding to EGR1 promoter leading to reduction in EGR levels	(Deckmann et al., 2010, 2012)	
(**18**)	Reduction of HDAC activity	Compound (**18**) is a carboplatin and 4-phenylbutyrate hybrid which presents an IC_50_ significantly lower than its reference compounds against A431 cells.	(Almotairy et al., 2017)	
N-(2′-Hydroxyphenyl)-2-propylpentanamide (**19**)	HMGB1 translocation from nucleus to cytoplasm in HeLa cells, probably resulted from HDAC inhibition	Compound (**19**) presented an activity against HeLa cells and IC_50_ value of 0.92 mM and increased intracellular levels of ROS after treatment with a concentration of 0.8 mM.	(Oca et al., 2018; Sixto-López et al., 2020)	HDAC1:153.78 μM HDAC6:>1000 μM HDAC8:>1000 μM
Luotonin A derivative (**20**)	Inhibition of HDAC 1 and 2	Compound (**20**) presented an antiproliferative activity against HeLa cells identical to Luotonin A, its precursor; however, the selective index was 4.4 times superior for the synthesized compound compared to Luotonin A. Compound (**20**) treatment also resulted in induction of p53 protein and G1 arresting cell cycle.	(Venkatesh et al., 2015)	HDAC1:2.96 μM HDAC2:6.40 μM
**FDA-approved drugs**				
Vorinostat (SAHA)	HDAC pan-inhibitor	ED_50_ against cervical cancer (CaSki, SiHa, and HeLa cells) ranging from 0.5 to 5.1 μM. In HeLa cells vorinostat was able to downregulate several proteins, such as ALDR, HSPB1, IF5A1, and PGAM1. Also in HeLa cells, a reduction in the mRNA levels of HPV18 E6 and E7 and transcription of both genes were reported, suggesting that vorinostat is a useful drug for cervical cancer.	(Noriyuki et al., 2007; Lin et al., 2009; He et al., 2014; Yunfei et al., 2015)	HDAC1:0.06 μM HDAC2:0.3 μM HDAC3:0.02 μM HDAC4:>10 μM HDAC5:>10 μM HDAC6:0.009 μM HDAC7:>10 μM HDAC8:>0.8 μM HDAC9:>10μM HDAC10:>0.03 μM
Belinostat (PXD101)	Major inhibition of HDAC1	Belinostat was able to significantly reduce the tumor growth in an *in vivo* experiment using nude mice with either human ovarian or colon tumor in a 7-days treatment.	(Plumb et al., 2003)	HDAC1:34 nM HDAC4:9,850 nM HDAC6:27 nM HDAC8:353 nM HDAC11:25,000 nM
Panobinostat	HDAC pan-inhibitor	The EC_50_ in HeLa cells for panobinostat was 0.1 μM. Against cervical cancer cells, this drug was able to reduce the cell viability in a dose- and time-dependent manner. Panobinostat increased reactive oxygen species levels inside the cells, disrupted mitochondrial membrane potential, increased levels of p21 and caspase-9, and reduced the expression of Bcl-xL genes.	(Khan et al., 2008; Wasim and Chopra, 2016)	HDAC1:3 nM HDAC2:3 nM HDAC3:4 nM HDAC4:12 nM HDAC6:61 nM HDAC7:14 nM HDAC8:248 nM HDAC9:3 nM

*HDAC, histone deacetylase*.

#### Effects of FDA-Approved Drugs Against Cervical Cancer

Not all approved drugs were tested against cervical cancer lines. In this section, we will discuss the literature data found in the last 20 years regarding HDACi approved drugs and their effects in cervical cancer.

#### Vorinostat (SAHA)

Vorinostat (Figure 5) is a hydroxamic acid derivative known as pan-inhibitor of HDACs. Structurally, X-ray crystallography experiments revealed that hydroxamic acid subunits interact directly with the zinc atom present in the active site of the HDAC enzymes (Finnin et al., 1999). Some studies have suggested the mode of interaction of vorinostat with HDAC class II using molecular modeling (Tambunan and Wulandari, 2010; Tambunan et al., 2011). Compared to other hydroxamic acid derivatives (i.e., TSA), vorinostat exhibits appropriate pharmacokinetic, low toxicity, and effectiveness (Noriyuki et al., 2007). It has been reported that ED_50_ of vorinostat against cervical cancer using different cell lines, including CaSki, SiHa and HeLa, ranged from 0.5 to 5.1 μM (Noriyuki et al., 2007; Lin et al., 2009).

Proteomic analysis of HeLa cell culture treated with vorinostat revealed that four proteins were downregulated after the treatment: aldose reductase (ALDR), heat-shock protein beta-1 (HSPB1), eukaryotic translation initiation factor 5A-1 (IF5A1), and phosphoglycerate mutase 1 (PGAM1). The latter is one of the main enzymes involved in glycolysis that is upregulated in the carcinogenic process. Not only HeLa but also CaSki cells have increased the expression of PGAM1 (He et al., 2008). A study has associated the upregulation of PGAM1 with immortalization (Evans et al., 2005). In HeLa cells, vorinostat also induces apoptosis through a series of mechanism including mitochondrial membrane potential, caspase activation, and PARP cleavage. In addition to that, the drug increases reactive oxygen species (ROS) and decreases the levels of glutathione (GSH) and thioredoxin (You and Woo, 2014). The relationship between human papillomavirus (HPV) infections and cervical cancer is widely described. The treatment of HeLa cells with vorinostat reduced the mRNA levels of HPV18 E6 and E7 and transcription of both genes. The high levels of acetylation on HPV18 promoter downregulates E6 and E7, suggesting voronistat as a useful drug for cervical cancer (He et al., 2014).

A study with HPV-18 in organotypic raft cultures of primary human keratinocytes demonstrated that vorinostat was able to inhibit viral DNA amplification significantly, and more than 30% of the treated cells underwent apoptosis. 5 μM vorinostat reduced E6 and E7 activity resulting in an elevation of p53, probably being the cause for the inhibition of viral DNA amplification. HPV-infected cells have shown to be more sensitive to vorinostat than uninfected ones (Banerjee et al., 2018).

#### Belinostat

Belinostat (Figure 5) (also known as PXD101) is a potent HDAC inhibitor approved by the FDA in 2014 to treat peripheral T-cell lymphoma (Foss et al., 2015). HDAC from HeLa cell nuclear extracts, mainly HDAC1 and HDAC2, were evaluated after previous treatment with belinostat. In this condition, the value of IC_50_ for HDAC inhibition was 51 nM. Enzymatic assays with different HDAC isoforms were also evaluated and shown for belinostat values of IC_50_ of 34 nM (HDAC1), 353 nM (HDAC8), 9850 nM (HDAC4), 27 nM (HDAC6), and 25000 nM (HDAC11) (Li et al., 2016). In fact, HDAC1 seems to be one of the main targets of belinostat since HeLa cell knockdown for HDAC1 decreases its sensitivity to belinostat (Dejligbjerg et al., 2008). The belinostat treatment was able to increase acetylation of histone H4 in several tumor lines at a dose-dependent manner, including HeLa cells. An *in vivo* experiment using nude mice with either human ovarian or colon tumors at a dose of 40 mg/kg/day through i.p. route during 7 days reduced significantly the tumor growth with no acute observable toxicity (Plumb et al., 2003).

#### Panobinostat

Panobinostat (Figure 5) is an oral HDAC inhibitor with an anticancer effect against several lines of cancer cells. It is approved for the treatment of relapse or refractory multiple myeloma (Kyriaki et al., 2018). Using recombinant enzymes, it was found that panobinostat has a role as pan-inhibitor, being more potent than vorinostat and belinostat. The IC_50_ values found for panobinostat were in the nanomolar scale: 3 (HDAC1), 3 (HDAC2), 4 (HDAC3), 12 (HDAC4), 61 (HDAC6), 14 (HDAC7), 248 (HDAC8), and 3 (HDAC9). In the cell proliferation assay using HeLa cells, the EC_50_ value of panobinostat was 0.1 μM. Lysate of HeLa cells exposed to twice the EC_50_ concentration for 24 h has induced its histone H3 and H4 acetylation (Khan et al., 2008). In cervical cancer cells, it was found that panobinostat increases histone H3 acetylation and diminishes the cellular viability in a dose- and time-dependent manner. Interestingly, for SiHa cells the treatment of panobinostat arrested the G2/M phase, while for HeLa the G0/G1 phase of the cell cycle was arrested. Several mechanisms of apoptosis were described for panobinostat, such as the ability to raise reactive oxygen species levels inside the cells, disrupt the mitochondrial membrane potential, increase the levels of p21 and caspase-9, and reduce the expression of Bcl-xL genes (Wasim and Chopra, 2016).

#### Association of Anticancer Drugs With HDACi for Cervical Cancer

In general, the effectiveness of HDACi alone against solid tumors is inferior to that of the association with an anticancer drug. Therefore, combinations of drugs act synergically by increasing the anticancer activity and reducing adverse effects, since low doses can be used. Examples of associations of HDACi have been successfully described for kinase inhibitors (i.e., imatinib), proteasome inhibitors (i.e., bortezomib), topoisomerase I and II inhibitors (i.e., topotecan, doxorubicin), anti-tubulin drugs (i.e., paclitaxel), heat shock protein-90 inhibitors (i.e., geldanomycin), DNA-covalent ligands (i.e., cisplatin), and DNA methylation inhibitors (i.e., decitabine), among others. In this section, some limited examples will be presented to demonstrate the importance of this approach in the therapy.

A combination of panobinostat with topoisomerase's inhibitors (topotecan or etoposide) enhances apoptosis in both HeLa and SiHa cells. The induction of intrinsic apoptosis was mediated by several mechanisms involving the activation of the ERK pathway and inhibition of both NF-κB and PI3K/AKT pathways. In addition to that, high levels of ROS and mitochondrial injuries contribute to activate the apoptosis mechanism. For example, the association of panobinostat and topotecan increased the levels of ROS at 68.6 and 21.3% in HeLa and SiHa cells when compared to their respective single treatments (Wasim and Chopra, 2018). The synergism between HDACi and topoisomerase inhibitors can be explained based on HDACi's effect, which after hyperacetylation keeps the chromatin structure opened, allowing easy access to DNA by damaging agents (Nolan et al., 2008).

The association of vorinostat with doxorubicin improves the cytotoxic effects of the last one, demanding low doses of doxorubicin. Using HeLa, CaSki, and SiHa cell lines, it was possible to demonstrate that this combination treatment is able to induce apoptosis by activating caspase-3 and poly(ADP-ribose) polymerase cleavage and upregulating the Bad protein. Acetylation of p53 results in the transcriptional activation of target genes, such as the pro-apoptotic Bad protein (Lee et al., 2014).

The combination of HDACi and proteasome inhibitors has been described in the literature as a useful approach to treat cervical cancer. Proteasomes are responsible for protein degradation, which maintains the intracellular balance and acts on breakdown transcription factors and proteins involved in the cell cycle. Bortezomib was the first proteasome inhibitor approved by the FDA to treat multiple myeloma. This drug exhibits activity against several types of cancer, including human cervical carcinomas (Birle and Hedley, 2007). A combination of vorinostat and bortezomib induces apoptosis in HeLa cells at superior levels compared to the exposure of those separated agents. This effect is associated with the augmentation of the Bax/Bcl-2 expression ratio, caspase-3 activation, and reduction of NF-κB and Akt expression levels in HeLa cells (Jiang et al., 2010).

Another study combining bortezomib with the pan-HDACi vorinostat and trichostatin A revealed that cervical cancer cell lines are more responsive than HPV-negative cervical cancer. For cervical cancer cells, a high expression of HDAC1, HDAC2, and HDAC6 was found. The bortezomib treatment reduced cell viability of CaSki, SiHa, and HeLa cell lines at nanomolar concentrations and increased the level of p53. This cytotoxic effect was limited in those non-transformed cervical cancer cells. The combination of vorinostat or trichostatin A with bortezomib induces apoptosis selectively in HPV-positive cervical cancer cells. *In vivo* studies were performed in order to evaluate the effect of drugs combination in the treatment of xenograft tumor using immunodeficient female mice. In this experiment, the single treatment with bortezomib or trichostatin A was able to reduce tumor growth and expand the animal's survival, but superior effects were observed after the association of both drugs. Immunoblot analysis showed a pronounced poly(ADP-ribose)polymerase cleavage in the association compared to the single treatment (Lin et al., 2009; Huang et al., 2015).

In ovarian cancer cells, bortezomib enhances cytotoxicity through antigen-specific CD8+ T cells, which allows better immune response (Chang et al., 2012; Kim et al., 2013). *In vivo*, the combination of vorinostat and bortezomib improves even more the specificity of the immune system against the tumor, making cancerous cells more susceptible to antigen-specific CD8+ T cells than those treated with isolated drugs (Huang et al., 2015).

The combination of treatments using cisplatin and vorinostat acts synergically against HeLa cells, being more active than the isolated treatment. The apoptosis of HeLa cells was induced by the activation of caspase-3 and inhibition of expression of Bcl-2 and x-linked inhibitor of apoptosis protein (XIAP). The relaxation of chromatin induced by HDACi could partially explain the increased effect when compared to the treatment with a single drug (Jin et al., 2010).

A combination of valproic acid with the experimental compound VE465, an aurora kinase inhibitor, has shown synergism in the cervical cancer cell line ME180. Aurora kinase plays an important role in tumorigenesis, and its overexpression has been detected in several human cancers. The IC_50_ values of valproic acid for CaSki and ME180 cell lines were 4 and 5.1 mM. The IC_50_ value was reduced up to 1.9 mM after combining both compounds (Li et al., 2013). A better effect was also observed after the combination of wortmannin, a phosphatidylinositol 3-kinase inhibitor, and the HDACi sodium butyrate. For this combination, an increased expression of p21, p27, and p53 was detected in HeLa cells previously treated with the compounds. Furthermore, activation of caspase 3 and 9 and high levels of PARP cleavage were observed (Park et al., 2006).

A phase I trial revealed that only magnesium valproate at doses of 20 and 40 mg/kg could inhibit HDAC activity leading to high levels of acetylation in tumor tissues (Chavez-Blanco et al., 2005). Therefore, using a drug-repurposing strategy, a combination of hydralazine and magnesium valproate (TRANSKRIP®) was proposed as a new anticancer agent (Dueñas-Gonzalez et al., 2014). Hydralazine is an inhibitor of the DNA methylation enzyme (DNMT). Phase II clinical trials in patients with cervical cancer described that hydralazine–valproate treatment associated with standard treatment with cisplatin chemoradiation has improved efficacy when compared to an isolated treatment (Mani et al., 2014). Preliminary data from a phase III study using a combination of hydralazine–valproate, cisplatin, and topotecan demonstrated a progression-free survival using the combining therapy compared to standard treatment. In this study, the median PFS of the control group was completed in 4.6 months and the experimental arm in 7.6 months. In order to be considered in the evaluation of the results, the patients had to complete their first cycle of protocol therapy, after which they underwent a repeated evaluation of the disease before starting the second cycle. PFS was defined as the minimum time until clinical progression, death, or data from the last contact and measured from the period data, in which the Kaplan–Meier procedure was used for all patients. Thus, they demonstrated that the drugs are capable of increasing the PFS in patients with cervical cancer with increased but manageable expenses; however, this study was discontinued (Coronel et al., 2011).

The association of valproic acid (VPA) and trans-retinoic acid was able to activate the dormant tumor suppressor gene RARß2, inhibiting both *in vitro* and *in vivo* cervical cancer growth. *In vitro*, the upregulation of RARß2 gene expression was found to be up to 90-fold. Moreover, upregulation of p21 and p53 and activation of the PI3K/Akt pathway and diminishing of p-Stat3 levels were reported for this combination (Feng et al., 2012). *In vivo* experiments were also carried out in a xenograft model using human squamous cell carcinoma. In this assay, the combination of valproic and trans-retinoic acids increased the levels of tumor suppressor genes, as well as RARß2, p53, p21^CIP1^, and E-cadherin. Levels of loricrin and involucrin were also upregulated, contributing to apoptosis and reduction in tumor size (Feng et al., 2013).

#### HDACi Effectiveness Against HPV Infections

Regardless of several studies having demonstrated the efficacy of HDACi in cervical cancer cell lines, little is known about their impact in the HPV life cycle and its outcome on HPV infections. The effectiveness of HDACi to HPV infections, which is the major risk factor for cervical cancer, is still controversial. Previous studies have demonstrated that when HeLa cells were treated with SAHA, HPV18 E6 and E7 mRNA and protein levels were reduced, and HPV18 promoter activity was decreased, suggesting that SAHA inhibited the transcription initiation of HPV18 E6 and E7 genes (He et al., 2014). Also, the authors observed a correlation between histones' deacetylated sites and the downregulation of mRNA HPV18 E6 and E7 when HeLa cells were treated with SAHA. Another study demonstrated that hydralazine and valproate could be safely administer to HPV-related tumors, such as cervical cancer, because they do not increase viral oncoprotein expression and also valproate-induced hyperacetylation of p53 protein, protecting the infected cells from their degradation by E6 (La Cruz-Hernández et al., 2007). A recent study investigates that two other HDAC inhibitors, belinostat and panobinostat, also inhibited viral DNA amplification and caused apoptosis (Banerjee et al., 2018). They verified a reduction of HPV18 E6/E7 protein levels and the ability of E6 to destabilize p53, which was promoted by the vorinostat treatment. The *in vitro* experiments using a synergistic formulation of curcumin, epicatechin gallate, resveratrol, and tricurin demonstrated that it suppresses the HPV E6 gene expression and eliminates HPV+ in TC-1 (express E6/E7) and HeLa cells (HPV 18) and activates apoptosis (Mukherjee et al., 2017).

Although some studies pointed to HDACi as a promising therapy to cervical cancer, contributing to abrogation of HPV16 and HPV18 E6/E7 mRNA and protein levels, previous studies, using HDACi valproate (VPA), sodium butyrate (NaBut), and trichostatin A (TSA), suggested caution (Bojilova et al., 2016). This study highly demonstrated the transcription of the reporter gene under the control of the HPV-16 LCR in different cell lines, including SiHa and Hela cells, respectively HPV16 and HPV18. Bojilova et al. (2016) demonstrated that TSA inducted 2–4-fold increment in the HPV LCR-driven transcription of the luciferase reported in HaCaT cells, and the HPV-negative keratinocyte cell line was able to differentiate (unlike HeLa, SiHa, and BeWo cells). In the same direction, Johansson et al. (2015) verified that HDACi could increase histone acetylation on the HPV16 genome correlated with high HPV16 gene expression, causing a 2- to 8-fold induction of HPV16 early and late mRNAs in cervical cancer cells.

## Conclusions

HDACi has demonstrated enough potential to treat cervical cancer, alone or in association. It is not completely clear which HDAC inhibition is more prone to control cervical cancer cell proliferation; however, some studies suggest the importance of HDAC class I to reach this effect. In this review article, we have also demonstrated that the association of HDACi with anticancer drugs presents a combined effect, allowing the reduction of the anticancer dose and contributing to reducing the adverse effects associated with the treatment. Cervical cancer cells are more sensitive to associations of drugs than the use of HDACi alone. Few clinical trials were performed using HDACi for cervical cancer, but some studies involving drug association seem to be promising. HDACi can interfere simultaneously in several cellular mechanisms which include high levels in pro-apoptotic Bcl-2 family proteins; activation of caspase-3, -8, and -9; and modification on mitochondrial membrane potential and cytochrome c release. All these events activate cell apoptosis. In addition, HDAC9 also interferes in the expression of E-cadherin and beta-cadherin, altering the transcription of several oncogenes. High levels of p21 and p27 are also reported for HDACi, leading to alteration in the cell cycle. Although several studies have been performed with cervical cancer in HPV-positive cell lines, the effectiveness of HDACi to interrupt high-risk HPV E6 and E7 levels, which is a hallmark risk factor to trigger cervical cancer, is still controversial. Future studies should be conducted to better understand the therapeutic potential against cervical cancer associated with HPV.

## Author Contributions

All authors listed have made a substantial, direct and intellectual contribution to the work, and approved it for publication.

## Conflict of Interest

The authors declare that the research was conducted in the absence of any commercial or financial relationships that could be construed as a potential conflict of interest.
